# Fatty Acid Metabolism Disruptions: A Subtle yet Critical Factor in Adverse Pregnancy Outcomes

**DOI:** 10.7150/ijbs.103404

**Published:** 2024-11-04

**Authors:** Xiao-Yan Cao, Meng-Ying Li, Chang-Xiang Shao, Jia-Lu Shi, Tao Zhang, Feng Xie, Ting Peng, Ming-Qing Li

**Affiliations:** 1 Department of Reproductive Immunology, The International Peace Maternity and Child Health Hospital, School of Medicine, Shanghai Jiao Tong University, Shanghai 200030, People's Republic of China.; 2 Laboratory for Reproductive Immunology, Hospital of Obstetrics and Gynecology, Shanghai Medical School, Fudan University, Shanghai 200080, People's Republic of China.; 3 Department of Obstetrics and Gynecology, Shanghai Changning Maternity & Infant Health Hospital, East China Normal University, Shanghai 200051, People's Republic of China.; 4 Assisted Reproductive Technology Unit, Department of Obstetrics and Gynecology, Faculty of Medicine, Chinese University of Hong Kong, Hong Kong, People's Republic of China.; 5 Shanghai Key Laboratory of Embryo Original Diseases, Shanghai 200030, People's Republic of China.

**Keywords:** fatty acid oxidation, omega-3 fatty acids, oxidative stress, inflammation, pregnancy outcomes

## Abstract

The establishment and maintenance of pregnancy encompass a series of complex and high-energy-consuming physiological processes, resulting in a significant energy demand. Fatty acids, one of the most essential nutrients, play a crucial role in energy supply via oxidation and perform critical biological functions such as anti-inflammatory and anti-oxidant effects, which substantially impact human health. Disordered fatty acid metabolism can cause anomalies in fetal growth and development, as well as a range of pregnancy problems, which can influence the health of both the mother and the fetus. In this review, we innovatively explore the relationship between fatty acid metabolism abnormalities and pregnancy complications, emphasizing the potential of dietary interventions with polyunsaturated fatty acids in improving pregnancy outcomes. These findings provide important evidence for clinical interventions and enhance the understanding and practical application of health management during pregnancy.

## Introduction

The maintenance of metabolic homeostasis is fundamental to the success of pregnancy 1. Fatty acid oxidation (FAO) is essential during pregnancy since it is a highly efficient energy metabolism pathway. Approximately 30 molecules of ATP can be generated during the complete oxidation of a single glucose molecule. In comparison, one stearic acid molecule has the potential to produce up to 106 ATP molecules, illustrating the superior energy yield of fatty acids 2. Mounting evidence indicates a strong correlation between anomalies in fatty acid oxidation and a range of adverse pregnancy outcomes 3, 4. In addition, fatty acids and their derivatives, such as prostaglandins, have signaling roles that can affect pregnancy outcomes by regulating inflammatory responses. Pregnancy is a state of chronic inflammation, with a mild inflammatory response required for regular pregnancy maintenance 5. An imbalance in the ratio of n-3 to n-6 polyunsaturated fatty acids might result in an overactive inflammatory response, adversely affecting the health of the mother and the fetus 6. N-3 polyunsaturated fatty acids are acknowledged for their anti-inflammatory and anti-oxidant characteristics, which aid in preserving lipid metabolism homeostasis in the human body 7. According to relevant studies, an appropriate supplementation of n-3 polyunsaturated fatty acids during gestation can promote fetal growth while lowering the risk of certain adverse pregnancy complications, regardless of whether there are any risk factors for pregnancy complications 8, 9. In conclusion, fatty acid metabolism and pregnancy outcomes have a complex and intimate link.

## Fatty Acid Metabolism

### Fatty Acids and Their Derivatives

Fatty acids (FAs) are carboxylic acid compounds with hydrocarbon chains linked to a carboxyl group. They can be represented by the standard structural formula CH3(CH2)nCOOH 10. FAs can be categorized according to their structural attributes. First, FAs are classified into different categories based on the number of carbon atoms in their carbon chain. These categories include short-chain fatty acids (SCFAs, C1-6), medium-chain fatty acids (MCFAs, C7-12), long-chain fatty acids (LCFAs, C13-22), and very long-chain fatty acids (VLCFAs, >C22) 11. Second, FAs can be classified as saturated (SFAs) or unsaturated (USFAs) based on the location of double bonds in the carbon chain. SFAs have no double bonds. USFAs can be further split into monounsaturated fatty acids (MUFAs) with a single double bond and polyunsaturated fatty acids (PUFAs) with two or more double bonds 10. PUFAs can then be categorized based on the position of the double bonds, including n-3, n-6, n-7, and n-9 clusters 10. In addition, FAs can be classified based on their sources into essential fatty acids (EFAs), which must be obtained from diets, and non-essential fatty acids (NEFAs), which the body can synthesize 10. Beyond serving as energy substrates, FAs play critical roles in cell membrane formation, signal transduction regulation, gene expression modification, and lipid mediators production 7, 10.

**Figure 1:**
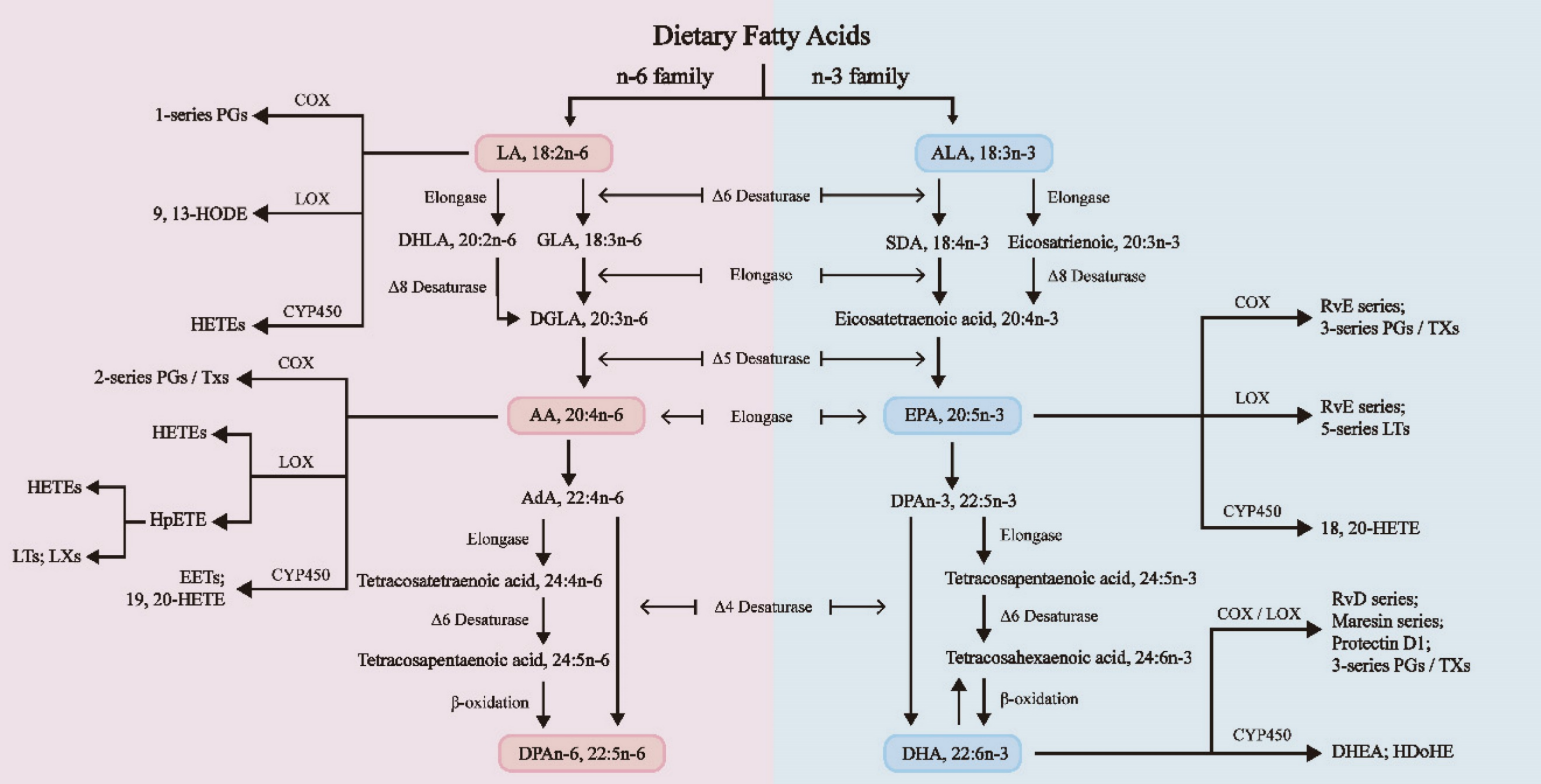
The biosynthesis pathways of n-3 and n-6 PUFAs 14. N-3 and n-6 PUFAs undergo a series of biosynthetic processes utilizing the same desaturases and elongases. Stearic acid is a lipid mediator from C18 polyunsaturated fatty acids, such as ALA or LA. Eicosanoids are formed from C20 polyunsaturated fatty acids, such as DGLA, ARA, or EPA. Docosanoids are synthesized from C22 polyunsaturated fatty acids, such as DPAn-3 and DHA. The n-3 and n-6 PUFAs undergo metabolism via LOX, COX, and CYP pathways. These processes can transform EPA and DHA into specialized proresolving mediators (SPMs), such as resolvins, maresins, and protectins, which possess anti-inflammatory characteristics 44. AA: Arachidonic acid; AdA: Adrenal acid; ALA: α-Linolenic acid; DHA: Docosahexaenoic acid; DHLA: Dihydrolipoic acid; DHEA: Docosahexaenoyl ethanolamide; DGLA: Dihomo-γ-linolenic acid; DPA: Docosapentaenoic acid; EET: Epoxyeicosatrienoic acid; EPA: Eicosapentaenoic acid; GLA: γ-Linolenic acid; HDoHE: Hydroxydocosahexaenoic acid; HETE: Hydroxy eicosatetraenoic acid; HODE: Hydroxy octadecadienoic acid; HpETE: Hydroperoxy eicosatetraenoic acid; LA: Linoleic acid; LT: Leukotriene; LX: Lipoxin; PG: Prostaglandin; Rv: Resolvin; SDA: Stearidonic acid; TX: Thromboxane.

As shown in **Figure 1**, FAs undergo diverse chemical changes to derive a range of bioactive molecules with numerous biological activities. Among them, n-3 and n-6 long-chain polyunsaturated fatty acids (LC-PUFAs) extensively affect human physiological health 12. Linoleic acid (LA) and alpha-linolenic acid (ALA) are the primary PUFAs in Western diets and are also the two primary dietary EFAs for humans, mainly sourced from vegetable oils 13. These crucial fatty acids are mainly converted in the liver through enzymatic elongation and desaturation reactions into longer, highly unsaturated FAs. ALA is a precursor for n-3 PUFAs, which are then turned into eicosapentaenoic acid (EPA) and docosahexaenoic acid (DHA). On the other hand, LA is a precursor for n-6 PUFAs, which are then converted into arachidonic acid (AA) and gamma-linolenic acid (GLA) 14. DHA, EPA, and AA are three "star" PUFAs participating in various physiological and pathological processes 10. It is crucial to acknowledge that the projected conversion rate of ALA to EPA is just 8% to 12%, and the conversion to DHA is considerably lower, at just 1% 15. Therefore, it is vital to regularly consume a moderate quantity of fish and fish oils, as they are abundant in EPA and DHA 12.

N-3 and n-6 PUFAs regulate inflammation and oxidative stress responses by engaging in enzymatic pathways that involve cyclooxygenases (COX), lipoxygenases (LOX), and cytochrome P450 (CYP450). These pathways lead to the synthesis of various eicosanoids, each serving distinct biological functions. These mechanisms are interconnected with the establishment of the menstrual cycle, the implantation of the embryo, the pregnancy maintenance, and the labor process 16.

### Fatty Acid Oxidation and Peroxidation

Normal fatty acid metabolism is crucial for maternal and fetal health during pregnancy 17. The energy demand during pregnancy is immense, and fatty acid oxidation is a key energy source for cells. However, fatty acid peroxidation is considered a major molecular mechanism involved in oxidative damage to cell structures and leads to cell death 18. Under pathological conditions, oxidants reacting with fatty acids can result in lipid peroxidation, producing cytotoxic substances that trigger inflammation and oxidative stress 19. This imbalance between oxidation and antioxidant defenses is associated with various pregnancy complications 19. Therefore, understanding the differences between these two processes and their roles during pregnancy is essential for preventing and managing pregnancy-related diseases.

#### β-Oxidation

**Figure 2:**
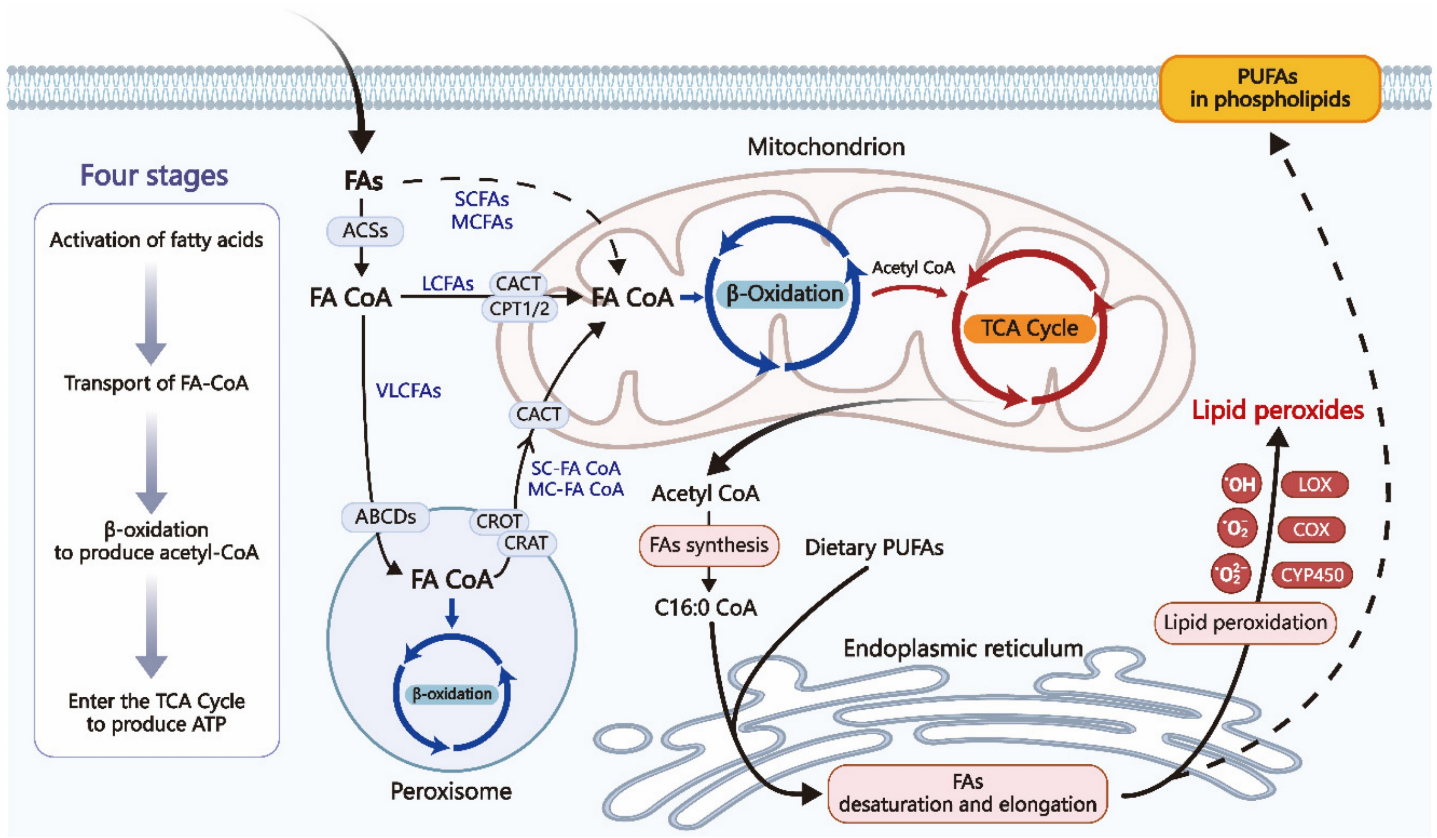
Four main stages of fatty acid β-oxidation. VLCFAs start β-oxidation in peroxisomes, while MCFAs and LCFAs initiate β-oxidation in mitochondria. Dietary PUFAs and endogenously produced FA precursors can undergo conventional elongation and desaturation processes, forming compounds that can be integrated into cellular membranes. Phospholipase A2 can break down phospholipids in cell membranes, releasing PUFAs. Lipid peroxidation is when oxidizing agents, such as free radicals and reactive oxygen species (ROS), react with FAs with double bonds, especially PUFAs. Enzymatic or non-enzymatic processes can both mediate this process. VLCFA: Very Long-Chain Fatty Acids; MCFA: Medium-Chain Fatty Acids; LCFA: ong-Chain Fatty Acids; ACS: Acyl-CoA Synthetase; FA-CoA: Fatty Acyl-CoA; ABCD: Very Long-Chain Acyl-CoA Transporter; CPT: Carnitine Palmitoyl Transferase; CAT: Carnitine Acylcarnitine Translocase; C16:0-CoA: Initial product of fatty acid synthesis.

FAs are among the most prevalent organic acids in biological systems. Except for brain tissue, the majority of tissues in the body are capable of oxidizing fatty acids. Fatty acid β-oxidation in mitochondria is an essential process for cellular energy production. When glucose supply is limited, fatty acids serve as a significant energy source during both postabsorptive and fasted states 20. However, even when glucose is abundant, FAO remains the primary energy source for high-energy-demand organs such as the heart, skeletal muscles, and kidneys 20. As described in **Figure 2**, β-Oxidation consists of four stages: fatty acid activation, fatty acid transport to the mitochondria, β-oxidation leading to the production of acetyl-CoA, and the complete oxidation of acetyl-CoA in the tricarboxylic acid (TCA) cycle, finally ending in the liberation of a substantial quantity of ATP 10.

After fatty acids from the diet and adipose tissue reach target cells, SCFAs and MCFAs can pass through the plasma membrane via passive diffusion. LCFAs are actively transported across the plasma membrane by specific protein carriers. FAs must first be catalyzed by acyl-CoA synthetase (ACS) to form acyl-CoA before oxidation. LCFAs are esterified by long-chain acyl-CoA synthetase (LACS) located at the plasma membrane of adipocytes, the outer mitochondrial membrane of skeletal muscle, and microsomes in the liver, while SCFAs and MCFAs are activated within the mitochondrial matrix 21, 22. Carnitine palmitoyltransferase I (CPT1) converts long-chain acyl-CoA into acylcarnitine using carnitine. The acylcarnitine molecule traverses the inner mitochondrial membrane using the carnitine-acylcarnitine translocase (CACT). Carnitine palmitoyltransferase II (CPT2) converts acylcarnitine back to acyl-CoA in the mitochondrial matrix, and then the translocase transports carnitine back to the cytosol. The acyl-CoA esters undergo recurrent cleavage to generate acetyl-CoA, which is subsequently utilized in the TCA cycle to make reducing equivalents for oxidative phosphorylation 10.

The β-oxidation mechanism also exists in peroxisomes, where the oxidized substrates are very long-chain fatty acids. Their oxidation produces short-chain acyl-CoA and medium-chain acyl-CoA, which are transported across the peroxisomal membrane by carnitine O-acetyltransferase (CRAT) and carnitine O-octanoyltransferase (CROT), respectively, and then enter the mitochondria through the CACT for complete oxidation 23.

#### Fatty Acid Peroxidation

Reactive oxygen species (ROS) are crucial for properly executing maternal reproductive functions, including oocyte maturation, ovulation, and embryo implantation 24. However, excessive ROS can lead to fetal developmental disorders and increase the risk of pregnancy complications such as miscarriage, intrauterine growth restriction, preeclampsia, preterm birth, and gestational diabetes 24. Lipid peroxidation occurs when oxidants, such as ROS, interact with FAs containing carbon-carbon double bonds, particularly polyunsaturated fatty acids 25. The more double bonds there are, the higher the susceptibility of lipid peroxidation 12. This interaction includes the process of hydrogen atom removal from carbon and oxygen, resulting in the formation of lipid peroxyl radicals and hydroperoxides 25. In addition, lipid peroxidation can be facilitated by enzymatic processes involving COX, LOX, and CYP450 26. The products of fatty acid peroxidation include both cytotoxic and inflammatory capabilities, as well as cytoprotective and anti-inflammatory features. The specific effects depend on the type of fatty acid (such as n-3 or n-6) and the pathways involved (enzymatic or non-enzymatic) 11. Oxidative stress is characterized by an imbalance between the production of oxidants and the cellular antioxidant capacity 24. Normal human pregnancy is considered a state of enhanced oxidative stress, which plays a significant role in embryonic development and implantation, as well as fetal growth and delivery 27. However, there is compelling evidence indicating that women with various pregnancy complications often experience excessive levels of oxidative stress and increased lipid peroxidation in their placentas 28, 29. Due to the intricate alterations in immunological responses during pregnancy, the placenta is recognized as a major producer of ROS. Imbalances in the regulatory mechanisms that govern oxidative stress and antioxidant defenses can lead to a spectrum of complications during pregnancy 30.

## Fatty Acid Metabolism in Pregnancy

### Embryo Implantation

Embryo implantation is a complex process in which a blastocyst, capable of implantation, interacts with a receptive endometrium. This process involves the embryo's location, adhesion, and invasion 31. Endometrial receptivity must be appropriately established for successful implantation and pregnancy maintenance. The regulation of this process is intricately controlled by ovarian steroid hormones and requires the coordinated interaction of multiple cells and molecules 32. With the development of lipidomic, lipid molecules, particularly fatty acids, have begun to attract more attention among the diverse molecules involved in this process 33. The establishment of endometrial receptivity demands morphological and histological changes in the endometrium, which depend on the metabolism of glucose and fatty acids to obtain enormous energy and material resources 34. PUFAs and their derivatives can regulate endometrial receptivity through multiple pathways throughout implantation. We will elaborate on these aspects in the subsequent sections.

#### Important Energy Source

The formation of endometrial receptivity involves multiple morphological and histological changes, including inhibition of epithelial cell proliferation, remodeling of epithelial cells, and metaplasia of stromal cells 35. These processes are dependent on glucose and fatty acid metabolism for energy supply to provide an adequate source of energy and material 34. In this series of processes, the degree of endometrial decidualization determines the extent of trophoblast cell invasion, subsequently affecting embryo implantation, placentation, and the maintenance of normal pregnancy 36. Decidualization involves complex cellular and molecular changes, and the differentiation of endometrial stromal cells (ESCs) has significant energy metabolic requirements 37, 38. Studies have shown that human ESCs rely on active FAO to achieve successful decidualization 37, and inhibition of CPT1 has been demonstrated to inhibit human ESC decidualization, highlighting the importance of FAs β-oxidation 39.

#### Regulation of Inflammatory Response

According to Mor et al., pregnancy can be described into three stages based on the immunological environment 5. The initial stage of pregnancy involves the blastocyst implantation and the placenta's formation and development 5. Endometrial stromal cells and infiltrating immune cells (including macrophages, dendritic cells, and natural killer cells) release a significant quantity of pro-inflammatory cytokines (including IL-6, IL-15, and TNF-α) and chemokines (including CXCL10, CXCL8, and CCL2) during this phase, resulting in a pro-inflammatory environment at the implantation interface. This stage is favorable for the attachment of the blastocyst and the breakdown and restructuring of the decidua by the trophoblast 40. It is crucial to emphasize that, unlike acute inflammation, the degree of the inflammatory response involved in the implantation process is finely regulated and is not associated with neutrophil infiltration 41. The localized pro-inflammatory state not only promotes the attachment of the blastocyst but also optimizes endometrial receptivity, consistent with meta-analyses indicating that local endometrial biopsy or scraping before IVF/ICSI-ET treatment can improve reproductive outcomes in women with recurrent implantation failure 42, 43. PUFAs are expected to significantly impact this process by regulating the equilibrium between pro-inflammatory and anti-inflammatory reactions during embryo implantation.

The primary function of n-6 and n-3 PUFAs in regulating inflammation is attributed to their metabolic products, which interact with the inflammatory cascade as bioactive substances, such as eicosanoids 44. Pro-inflammatory properties are demonstrated by eicosanoids that are derived from n-6 PUFAs, such as 2-series prostaglandins, 2-series thromboxanes, and 4-series leukotrienes 45, 46. LTB4 and PGE2 increase vascular permeability and recruit inflammatory cytokines and immune cells, facilitating blastocyst implantation into the endometrium 47-49. In contrast, eicosanoids derived from n-3 PUFAs, involving 3-series prostaglandins, 3-series thromboxanes, and 5-series leukotrienes, exhibit weaker pro-inflammatory properties. Therefore, it is probable that n-3 PUFAs exert anti-inflammatory effects through various mechanisms, including: a) competition with AA for integration into membrane phospholipids and competing enzymatic pathways, thereby reducing pro-inflammatory eicosanoids derived from AA 44; b) The phosphorylation of the inhibitory subunit IκB of NFκB is inhibited by EPA and DHA, resulting in decreased activation of the pro-inflammatory transcription factor NFκB 50; c) DHA binds to the G protein-coupled receptor GPR120, which is highly expressed on inflammatory cells such as macrophages, thereby inhibiting the pro-inflammatory signaling pathways mediated by TLR2/3/4 and TNF-α 51; d) n-3 PUFAs suppress the activation of the NLRP3 inflammasome via GPR40 and GPR120-dependent pathways, essential for alleviating several pregnancy problems 52; e) promotion of M2 macrophage polarization by EPA and DHA to exert anti-inflammatory effects 7. Additionally, eicosanoids derived from EPA, DHA, and AA through LOX and COX pathways have garnered increasing attention for their role as SPMs in suppressing inflammatory responses. SPMs may mediate the resolution of pro-inflammatory signals, thereby controlling inflammation within reasonable boundaries 53. Dysregulation of the pro-inflammatory and anti-inflammatory mechanisms may lead to various pregnancy complications.

A quantitative analysis of plasma eicosanoid concentrations in 90 pregnant women revealed that the relative concentration of eicosanoids is highest during early pregnancy, with LA being the most abundant precursor 54. Considering the mechanisms described above, it is reasonable to speculate that the conversion of AA during pregnancy is quantitatively more active than that of EPA, leading to a higher ratio of n-6/n-3 PUFAs, presenting a pro-inflammatory state at the endometrial implantation interface, ultimately promoting successful pregnancy. Consistent with this, studies have found that women with a higher ratio of ALA/LA have higher embryo implantation rates than those with a lower ratio 55, 56.

The fine-tuning of pro-inflammatory and anti-inflammatory states runs throughout pregnancy 5. The pro-inflammatory states in early and late pregnancy facilitate embryo implantation and fetal delivery, while the anti-inflammatory state in mid-pregnancy supports rapid fetal growth 5. However, regardless of whether it is a pro-inflammatory or anti-inflammatory state, the relative balance between the two is always critical. Excessive inflammatory responses may lead to various pregnancy complications. We will elaborate on this in the following sections.

#### Regulation of Uterine Prostaglandins (PGs) Signaling

PUFAs are direct precursors to PGs 57. Variations in dietary PUFAs may influence PGs signaling in the uterus of animal models, which is considered another potential mechanism by which FAs affect embryo implantation 58. Among the various PGs, PGE2 and PGF2α are the two most critical molecules. During the window of implantation (WOI), the concentrations of PGE2 and PGF2α in human endometrial fluid, along with COX-2 levels in the endometrial epithelium, increase, collectively promoting a receptive endometrial state 58. Female mice that lack either Cytosolic Phospholipase A2 (cPLA2) or COX-2 experience a shortage in prostaglandin synthesis, which ultimately leads to the failure of implantation. However, exogenous supplementation with PGE2 and carbonyl prostaglandin (cPGI) can ameliorate this deficiency 59. Patients experiencing recurrent implantation failure (RIF) have shown a notable decrease in the levels of COX-2 and cPLA2α in the endometrial fluid 60. Vilella et al. suggested that the levels of PGE2 and PGF2α in the endometrial fluid 24 hours before embryo implantation could serve as potential non-invasive biomarkers for assessing endometrial receptivity and predicting pregnancy success 60. However, more high-level evidence is needed to support this. Furthermore, successful implantation also relies on the stable adhesion and robust invasion of trophoblast cells 61. Impaired invasion of extravillous trophoblasts (EVT) and insufficient remodeling of uterine spiral arteries are closely associated with pregnancy complications such as preeclampsia and intrauterine growth restriction 62. N-3 and n-6 PUFAs and their derivatives, particularly PGs, play significant roles in regulating the processes mentioned above. The following sections elaborate on these functions.

*Promotion of Trophoblast Adhesion.* During implantation, the endometrium secretes a significant amount of PGE2, which promotes the expression of prostaglandin E receptor 2 (EP2) on the blastocyst 63. PGE2 subsequently induces the phosphorylation of MAPK1/MAPK3 via EP2, upregulating the expression of proteins involved in cell-matrix interactions and adhesion (such as focal adhesion kinase and intercellular adhesion molecule-1), which in turn promotes the attachment of trophoblast cells to the extracellular matrix (ECM) 63. Additionally, PGE2 may increase the expression of integrin αvβ3 (ITG αvβ3), further promoting embryo adhesion 64. In epithelial cells, calreticulin (CALR) is regulated by exchange protein directly activated by cAMP 2 (EPAC2), which modulates Ca²⁺ influx and binds to intracellular Ca²⁺. The cAMP-PKA pathway can also induce COX-2-mediated PGE2 production, promoting trophoblast cell adhesion 65, while Ca²⁺ influx can trigger human trophoblast-like cell adhesion 66. Similarly, PGF2α promotes the adhesion of porcine and human trophoblast cells line HTR-8/SVneo to the ECM by activating FAK and MAPK signaling pathways 67, 68.

*Promotion of Endometrial Stromal Cell Decidualization.* The differentiation of endometrial stromal cells into decidual cells (DC) is crucial for female implantation and pregnancy. Dysregulation of decidualization is associated with pathological pregnancies, such as implantation failure, recurrent miscarriage, and late pregnancy complications 69. PGs can function as signaling molecules to control decidualization 38. In humans, 15-hydroxyprostaglandin dehydrogenase (15-PGDH) is the enzyme responsible for PG degradation, while the prostaglandin transporter (PGT) is a potential molecule involved in transporting PGs into cells. Blocking the enzyme 15-PGDH can enhance the process of ESC decidualization, leading to an increase in the expression of PGT, which helps in the intake of more PGE2 70. A study based on *in vitro* analysis of human endometrial stromal cells indicates that progesterone-induced decidualization is dependent on PG-induced cAMP signaling 71. Recent single-cell analyses of human decidual cells further indicate that the decidualization process is initiated by activating the progesterone-dependent PGE2/PTGER2/PKA axis 72. In addition, PGE2 and PGI2 can promote decidualization by increasing interstitial cAMP levels in the perivascular stroma through a paracrine mechanism 73.

*Promotion of Endometrial Angiogenesis.* Remodeling of the uterine spiral arteries within the first 20 weeks of pregnancy ensures that maternal blood is delivered to the placenta at a flow rate that meets the increasing demands for nutrients and oxygen by the fetus. In normal pregnancy, the process of remodeling the spiral arteries involves the migration and invasion of EVTs 74. EVT migration and invasion functions are strictly regulated, temporally limited to early pregnancy, and spatially confined to the endometrium, the upper third of the myometrium, and the associated spiral arteries 75. EVT exhibits all EP receptors (EP1-4), and the copious secretion of PGE2 by decidual cells predominantly binds to EP1 and EP4 receptors, facilitating EVT migration. The underlying mechanism may involve the activation of EP1 and EP4, which stimulate the expression of RAC1 and CDC42, two crucial mediators of EVT migration in early pregnancy 76. RAC1 can increase the activity of MMP-2 and MMP-9 by activating the MAPK8 pathway, commonly known as the JNK-dependent pathway. This activation helps break down the ECM in the decidua 76.

Angiogenesis in the endometrium during embryo implantation is crucial for maintaining normal pregnancy and improving fetal development. It involves numerous essential molecules, including vascular endothelial growth factor (VEGF), angiopoietin-like protein 4 (ANGPTL4), platelet-derived growth factor (PDGF), and platelet-activating factor (PAF) 77. VEGF robustly stimulates angiogenesis and increases vascular permeability 78. *In vitro* studies have shown that DHA stimulates the expression of VEGF mRNA and its protein secretion in HTR8/SVneo cells to promote angiogenesis 77, while other FAs (such as EPA, ARA, and OA) have a weaker stimulatory effect on ANGPTL4 release in HTR8/SVneo cells 77. The specific molecular mechanisms remain unclear.

PGE2 has been shown to induce vasodilation by binding to EP4 and activating endothelial nitric oxide synthase (eNOS) 79. In porcine endometrial stromal cells, PGE2 increased VEGF164 mRNA expression and VEGF secretion 80. Additionally, PGE2 can bind to EP2 on endometrial stromal cells, activating the epidermal growth factor receptor (EGFR)-phosphoinositide 3-kinase (PI3K) and extracellular signal-regulated kinase (ERK1/2) pathways, promoting the expression of chemokine receptor type 4 (CXCR4). CXCR4 subsequently interacts with chemokine 12 (CXCL12) secreted by the blastocyst 81. The CXCL12/CXCR4 signaling pathway increases the production and secretion of VEGF, and the upregulation of VEGF can further promote the synthesis of CXCL12 and CXCR4, creating positive feedback 82. PGF2α participates in the angiogenesis in an autocrine/paracrine manner, potentially involving the interaction of PGF2α with its receptors to stimulate the phosphorylation of MAPK1/3, leading to enhanced synthesis and expression of VEGF 83. PGI2 is also a vigorous vasodilator and has been reported to improve uterine vascular permeability by activating its nuclear receptor PPARδ 84.

### Fetal Growth and Development

The placenta facilitates the supply of nutrients and the removal of metabolic waste for the fetus 85. Therefore, the placental tissue, which acts as a bridge for maternal-fetal communication, necessitates a continuous and ample supply of energy and nutrients to fulfill the rapid growth requirements of the fetus 85. Glucose is widely recognized as the main energy source that sustains the growth and development of the embryo. However, glucose is not the sole energy source. During the last trimester of pregnancy, maternal lipid metabolism shifts predominantly to a catabolic state, resulting in significantly elevated triglycerides (TGs) and free fatty acids (FFAs) in maternal plasma 6. FFAs are transported to the fetal side through complex mechanisms 86. Studies have demonstrated that the enzymatic activity related to the oxidation of LCFAs is significantly elevated in human placental tissue 87. In addition, trophoblasts have also been observed to extensively oxidize FAs, demonstrating that the human placenta utilizes FAs as a significant metabolic source 4. FAs are widely utilized by the human placenta at all gestational ages, and any defects in the oxidation processes may hinder placental growth, differentiation, and function, thereby impairing fetal growth 4. Consistent with this opinion, the ablation of genes encoding enzymes involved in fatty acid oxidation (such as LCAD, VLCAD, and TFP) is associated with increased rates of intrauterine fetal demise and fetal growth restriction, underscoring the critical role of FAO in maintaining placental function and fetal growth 88, 89. Nonetheless, there currently needs to be more robust data evaluating the specific role of fatty acids as metabolic substrates for energy in fetal growth and development.

LA and ALA are two primary dietary EFAs predominantly found in plant oils. Their derivatives (AA, EPA, and DHA) can be obtained from animal-based diets 90. Dietary LA and ALA must be converted into LC-PUFAs mentioned above to exert their more refined biological effects 90. By the 26th week of gestation, the fetus can synthesize these LC-PUFAs from essential fatty acids 91. However, because the fetal body has limited activity of elongase and desaturase enzymes, the fetus mostly depends on the maternal supply of LC-PUFAs, which are carried through the placenta, for its growth and development 92. Insufficient levels of these EFAs throughout important stages of organ development in the embryo can result in severe and permanent effects on the growth and development of the fetus, especially in brain development 93. Bernard et al. assessed the plasma levels of LC-PUFAs in 985 mothers during weeks 26-28 of gestation and observed that the levels of LA and DHA were positively correlated with neonatal length 94. One high-quality evidence also indicates that supplementation with n-3 PUFAs reduces the risk of low birth weight infants 9. Elevated intake of LA may promote the production and transport of metabolic products such as AA to fetal tissues, where AA stimulates weight gain and fat tissue accumulation via the pro-inflammatory prostaglandin pathway 94. The mechanisms by which DHA promotes growth remain unclear, but possible explanations include increased levels of insulin-like growth factor-1 (IGF-1) and inhibition of osteoclastogenesis 94. Furthermore, it is crucial to consider the significance of inflammation and oxidative stress in the growth and development of the fetus 95. PUFAs and their derivatives may influence this process by modulating these factors. A nested case-control study found that compared to the appropriate for gestational age (AGA) control group, mothers who delivered small for gestational age (SGA) infants had higher levels of pro-inflammatory mediators in their plasma, while mothers who delivered large for gestational age (LGA) infants had lower levels 54. The strongest association with SGA was observed with AA-derived PGs, indicating that dysregulation of inflammatory processes may adversely affect fetal growth 54. The specific mechanisms require further investigation.

The fetal nervous system development begins early in pregnancy and continues throughout the third trimester and the first two years postnatally 96. LC-PUFAs, particularly DHA and AA, are vital in neurodevelopment 97. DHA and AA are the predominant LC-PUFAs in the brain and retina, and they, along with EPA and their derivatives, are involved in the formation and development of brain cells, promoting the synthesis of neuroproteins and the growth of neurons. They play significant roles in axonal extension and the formation of new synapses. Additionally, they are involved in processes connected to brain cognition and the generation of memories 97. DHA constitutes 50%-70% of the lipids in retinal cell membranes, primarily accumulating in phospholipids surrounding the protein rhodopsin, facilitating photoreceptor differentiation. When it receives signals, rhodopsin undergoes structural changes, which start a signaling cascade 98. Comprehensive studies indicate that nutritional supplements, including marine oil high in n-3 LC-PUFAs during pregnancy, can elevate maternal blood DHA levels and improve visual and cognitive functions in infants and children 99. However, other studies have yielded conflicting outcomes regarding these claims 100, and it is currently under investigation if the dietary LC-PUFAs promote newborn growth and neurocognitive abilities.

## Abnormal Fatty Acid Metabolism and Adverse Pregnancy Outcomes

During pregnancy, the maternal metabolic system undergoes significant changes to ensure an adequate supply of nutrients such as glucose, fatty acids, and amino acids, which are essential for fetal growth and development 101. The placenta, serving as a unique exchange organ between the mother and fetus, is responsible for transporting nutrients to the fetus and removing metabolic waste products 102. In recent years, disorders of deep placentation have been identified as a significant mechanism underlying various pregnancy complications 103. Deficiencies in placental function can lead to abnormal fetal development and pose risks to maternal and fetal health 103. Fatty acid metabolism influences normal placental function by regulating energy supply, oxidative stress, and inflammatory states, affecting different pregnancy stages.

### Spontaneous Abortion and Recurrent Spontaneous Abortion

Spontaneous abortion (SA) is defined as a miscarriage occurring before 20 weeks of gestation, with approximately 80% of cases happening in the early stages of pregnancy 104. Recurrent spontaneous abortion (RSA) is defined as two or more consecutive miscarriages occurring with the same partner 105. Despite various complex factors reported as potential causes of RSA, about 50% of its mechanisms remain unclear, posing significant clinical intervention and treatment challenges 105. RSA is often accompanied by metabolic changes, with critical pathways leading to a pro-inflammatory state and oxidative stress 106. These pathologic states can broadly affect oocyte development, blastocyst implantation, placental angiogenesis, and fetal development, ultimately resulting in miscarriage 107, 108.

In most cases of SA, there are defects in trophoblast proliferation, invasion, and remodeling of spiral arteries 109. These histological abnormalities can lead to excessive maternal blood entering the placental intervillous space, inducing oxidative stress at the maternal-fetal interface 109. A prospective cohort study involving patients with unexplained RSA found a correlation between FAs metabolic disorders and an increased number of observed miscarriages 110. Women with multiple miscarriages (≥5 times) exhibited increased lipolysis, decreased oxidation of MCFAs, poorer mitochondrial function, and elevated oxidative stress levels in the endometrium 110. Thus, it can be reasonably inferred that patients with RSA exhibit abnormalities in FAs metabolism, potentially leading to increased plasma PUFAs concentrations through mechanisms such as reduced FAO and increased lipolysis 110. PUFAs provide abundant substrates for lipid peroxidation, and these oxidized lipid mediators can be cytotoxic, acting as ligands for nuclear receptors involved in crucial metabolic and developmental pathways, adversely affecting maternal endothelial cell function, trophoblast invasion and placental function, and further increasing overall maternal-fetal oxidative stress levels 111. The oxidative stress state can also activate enzyme systems involved in fatty acid metabolism, including phospholipases and lipoxygenases, which in turn exacerbates the disturbances in fatty acid metabolism 106. A metabolomics study discovered elevated serum carnitine levels in the RSA group. Carnitine can promote the transport of FAs into the mitochondria for oxidation and also act as an antioxidant to scavenge excess free radicals 106. This indicates the presence of oxidative stress in RSA patients and a certain degree of recovery capacity. FAs transport and oxidation may also be promoted through compensatory mechanisms.

Inflammatory environment is another significant factor contributing to SA 112. A metabolomic study found that the ratio of C20:4n-6 to C20:5n-3 is significantly elevated in the placental decidua and appearance tissue of women experiencing early pregnancy loss, indicating an increase in inflammatory levels 113. Another study also indicated that total levels of PUFAs were associated with higher miscarriage risk, particularly total n-6 PUFAs 114. It is critical to recognize that the correlation between total plasma PUFAs concentrations and pregnancy outcomes may depend on the levels of total n-6 PUFAs and their pro-inflammatory properties, which exacerbate oxidative stress 115.

Abnormal metabolic pathways of FAs and their derivatives represent another potential mechanism contributing to SA. A metabolomic analysis revealed a notable upregulation of genes associated with AA metabolism pathways in RSA rats. These genes include COX-1, COX-2, PTGFR, and TBXA2R 116. Disrupted expression of COX genes and TBXA2R can cause an abnormal increase in PGs production via controlling the PLA2α/COX-2 pathway in endometrial stromal cells. This can lead to uterine contractions and ultimately result in RSA 116. Therefore, modulating AA metabolic pathways could serve as a potential therapeutic approach to relieve symptoms in patients with RSA. However, there is currently no high-quality evidence linking changes in individual n-6 PUFAs (such as AA) to miscarriage outcomes. Lipoxin A4 (LXA4), which is produced during the metabolism of AA, has anti-inflammatory properties. These properties include regulating cytokine levels, inhibiting leukocyte chemotaxis, and reducing reactive oxygen species formation 117. Both LXA4 and its biosynthetic enzymes decreased in women who have SA and in mice models of abortion 118, indicating that the precise effects of AA may necessitate a nuanced strategy. During the peri-implantation period, high levels of LXA4 may lead to early miscarriage, while low levels of LXA4 post-implantation may result in insufficient EVT invasion and placental dysfunction, adversely affecting fetal growth and pregnancy maintenance 119. The effects of dietary n-3 PUFAs in treating SA have been increasingly evaluated, as it can directly improve pregnancy outcomes in SA patients or indirectly ameliorate inflammation, oxidative stress, and even reverse mechanisms such as insulin resistance and thyroid dysfunction that contribute to the occurrence and progression of SA 120.

### Preeclampsia

Preeclampsia (PE) is a progressive multisystem disorder, diagnosed by sudden-onset hypertension (>20 weeks of gestation) and at least one other associated complication, including proteinuria, maternal organ dysfunction or uteroplacental dysfunction 121. Preeclampsia is a disease with a heterogeneous etiology involving multiple factors, mechanisms, and pathways 122. The classical "two-stage" theory posits that the first stage is a preclinical phase characterized by impaired remodeling of the uterine spiral arteries, resulting in placental ischemia and hypoxia. This results in the atypical secretion of various bioactive substances from trophoblast cells, including pro-inflammatory cytokines, reactive oxygen species, extracellular vesicles, anti-angiogenic agents, and fetal free DNA 123. In the second stage, these active factors enter the maternal bloodstream, activating a systemic inflammatory response and damaging vascular endothelium, leading to diverse clinical manifestations, including hypertension, liver and kidney dysfunction, thrombocytopenia, and coagulopathy 124. Abnormal fatty acid metabolism may lead to the development of PE by affecting energy supply, oxidative stress, and inflammatory responses.

According to the "two-stage" model, a crucial pathological feature of PE is the insufficient invasion of the EVT during early pregnancy 125. Early trophoblast differentiation to form the placenta and the invasion of EVT into the uterine decidua is highly dependent on energy. Insufficient energy supply can result in defects in the remodeling of uterine spiral arteries, leading to placental dysfunction and the progression of PE 126. Consistent with this perspective, studies have shown that in PE patients, levels of placental LCHAD mRNA, protein expression, and FAO activity are reduced 127. Additionally, the expression of crucial acyl-CoA dehydrogenases that catalyze FAO, such as HADHA, HADHB, and ACADV, is also reduced 128. FAO is essential for functional angiogenesis, particularly for endothelial cell proliferation 129. Recent research has demonstrated that CircHIPK3/miR-124 can regulate placental FAO activity and angiogenesis in EOPE through CPT1A, underscoring the importance of FAO in remodeling uterine spiral arteries 129.

Due to poor EVT invasion and insufficient remodeling of uterine spiral arteries, the placenta experiences repeated cycles of ischemia/reperfusion and hypoxia/reoxygenation, generating substantial ROS 130. When antioxidant mechanisms (often impaired in PE patients) fail to clear excess ROS promptly, the maternal environment experiences a state of oxidative stress higher than in normal pregnancies 131. At this point, the FFAs and metabolic intermediates accumulated during abnormal placental FAO undergo peroxidation with excess ROS. This process produces significant quantities of lipid peroxides, hydrogen peroxides, and aldehydes derived from lipid peroxidation. These substances are then transported to the mother's bloodstream, causing dysfunction in the endothelial cells and triggering excessive inflammation. Ultimately, this contributes to the advancement of preeclampsia 132. Excessive deposition of lipid metabolites can also damage mitochondria, further inhibiting FAO activity, promoting ROS production, accelerating apoptosis, and diminishing trophoblast invasion capacity 132. Supplementation with LC-PUFAs can effectively reduce placental oxidative stress, depending on the dosage and combination of LC-PUFAs. For instance, n-3 PUFAs can reduce the production of free radicals, promote angiogenesis, and improve hemodynamics 133. In contrast, n-6 PUFAs, particularly AA, appear to increase placental oxidative stress.

As previously mentioned, the maternal body undergoes necessary physiological inflammatory responses during pregnancy. However, when these inflammatory responses are excessively activated and progress to pathological inflammation, they can lead to immune imbalance and endothelial damage, promoting the occurrence and progression of pregnancy complications associated with PE 134. FAs may modulate inflammatory responses to either inhibit or promote the development of PE. HETE, a metabolite of AA, can result in poor placental perfusion 44. In PE patients, elevated levels of 5-, 8-, 12-, and 15-HETE have been observed at 20 weeks of gestation, with increased levels of 15-HETE and 12-HETE found in the placenta, and similar relationships noted in trophoblast cells 44. Additionally, excess 15-HETE has been detected in umbilical cord blood, potentially leading to constriction of the umbilical artery 44. LXA4 can both inhibit the production of TNF-α (a critical pro-inflammatory molecule driving the development of PE) to attenuate the inflammatory response 44 and promote nitric oxide expression to improve endothelial dysfunction in PE patients 135. Xu et al. discovered decreases in the levels of LXA4, its receptor formyl peptide receptor 2 (FPR2/ALX), and the enzymes responsible for producing LXA4 in the plasma of PE patients 136. SPMs may also contribute to symptom improvement in PE patients by participating in the resolution of inflammation 136. The NLRP3 inflammasome has emerged as a prominent area of research in the investigation of PE 137, while n-3 PUFAs can effectively inhibit the overactive inflammatory reactions by repressing the activation of the NLRP3 inflammasome 52. N-3 PUFAs may also exert their effects by reducing vascular responsiveness to vasoconstrictors such as angiotensin II and lowering blood viscosity 8.

The classification of early-onset preeclampsia (delivery < 34 weeks) and late-onset preeclampsia (delivery ≥ 34 weeks) facilitates a deeper investigation into their pathophysiological mechanisms 121. In early-onset preeclampsia (EOPE), the mechanisms are more closely related to placental dysfunction and damage, aligning with the traditional "two-stage" model 138, where clinical manifestations tend to be more severe compared to those in late-onset preeclampsia (LOPE), which is often considered a metabolic syndrome caused by maternal-fetal metabolic imbalance 139.

In terms of lipid metabolism, LC-MS/MS-based untargeted placental lipidomic studies have emphasized that unsaturated fatty acid-containing triglycerides may drive the occurrence and development of LOPE 140. Triglycerides can damage vascular endothelial cells and trigger inflammatory responses by affecting the maternal BMI 141. Placental factors do not appear to be the core cause of this subtype, and more precise molecular mechanisms require further investigation 142.

Therefore, to fully understand the metabolic crosstalk of PE within the entire body system, future studies need to consider grouping subjects by early-onset and late-onset types. Additionally, researchers should not isolate the metabolic levels of the placenta but rather conduct comprehensive inter-organ metabolomic flux analyses.

### Fetal Growth Restriction

Fetal growth restriction (FGR) is traditionally defined as a condition where the fetus's growth potential is compromised, with an estimated fetal weight less than the 10th percentile for gestational age 143. The etiology of FGR can be broadly categorized into maternal, fetal, and placental factors. Although the mechanisms leading to FGR development differ among these factors, they ultimately converge on placental insufficiency, characterized by impaired uteroplacental blood flow and altered placental function, resulting in fetal nutrient deficiency 143. During the critical window of placental development (primarily mid-pregnancy), insufficient blood flow and/or nutrient supply fails to satisfy the expanding nutritional requirements of the fetus, ultimately leading to developmental delays 144. The comprehensive metabolic analysis indicates that both maternal plasma and fetal umbilical cord blood exhibit dyslipidemia in pregnancies complicated by FGR 145. Moreover, several placental enzymes, such as endothelial lipase (EL) and lipoprotein lipase (LPL), which are crucial for supplying FAs to the developing fetus, show dysregulated expression in FGR 146. These findings collectively suggest the presence of abnormal FA metabolism in pregnancies with FGR, which will be discussed in a categorized manner.

Early-onset FGR (<32 weeks), is highly correlated with severe placental insufficiency 147. Insufficient EVT invasion affects the placental size, the formation of the villous tree, and the transfer of oxygen and nutrients, leading to FGR 147. Since early-onset FGR shares a very similar pathological mechanism with PE, and PE is often accompanied by FGR 148, there is also a widespread imbalance in energy metabolism, excessive oxidative stress, and inflammatory responses within the FGR placenta-fetal tissue 149. Thus, the more detailed association between early-onset FGR and FA metabolism can be referenced in the sections above regarding fetal growth and preeclampsia.

Late-onset FGR (≥32 weeks), accounts for 70-80% of FGR cases and shows fewer signs of placental disease. However, the fetal demand for oxygen and nutrients significantly increases. Despite this, maternal FA supply in FGR cases often exhibits abnormalities. Consequently, the placenta may employ compensatory mechanisms to sustain fetal growth and development, such as enhanced placental transport or upregulation of fetal FA synthesis 150. This is consistent with observations of upregulated mRNA expression of LPL, FA transport proteins (FATPs-1, -2, and -4), and FA binding proteins (FABPs-1 and -3) in the FGR placenta 151. Another research discovered increased expression of FATP6 and CD36 in the microvillous membrane of FGR placentas, along with elevated storage of LC-PUFAs in triglyceride fractions of FGR placentas 152. Further research has observed the accumulation of carnitine and increased FAO in the mitochondria of the term (gestational age >36.4 weeks) FGR placental trophoblast cells 153. Despite the existence of compensatory mechanisms, it is suspected that this may merely represent a compensatory upregulation, with FGR fetuses potentially still failing to obtain sufficient LC-PUFAs to meet their demands. Consistent with this hypothesis, a study found that umbilical cord blood of infants with FGR exhibited a deficiency in LC-PUFAs compared to the control group 150. The specific mechanisms regarding abnormal FA metabolism and late-onset FGR are currently unclear. NOTCH1 has recently been recognized as a key regulatory factor linking lipid metabolism dysregulation to placental growth, potentially playing an important role in the pathogenesis of late-onset FGR 154.

Classifying FGR into early-onset and late-onset categories facilitates the exploration of its pathological mechanisms and targeted interventions to improve pregnancy outcomes. It is necessary to adhere strictly to gestational age-based grouping for analysis in the future. At present, fundal height measurement remains the mainstream screening method for FGR, although its screening value before 24 weeks of gestation is limited 155. Given the close relationship between placental nutritional status and metabolic conditions, monitoring downstream metabolites may assist in more rapidly and accurately assessing the fetus's metabolic and developmental status regarding nutrient availability.

### Spontaneous Preterm Birth

Preterm birth (PTB) is universally defined as delivery before 37 weeks of gestation 156. However, the lower limit for defining PTB varies by country. Some developed countries and regions use 20 weeks or 24 weeks of gestation standards 156. Here, we focus exclusively on spontaneous preterm birth (SPTB). The exact etiology of SPTB remains unclear, but it may involve multiple factors such as excessive uterine distension, cervical incompetence, vascular diseases, chorioamnionitis, and placental dysfunction 156. In recent years, the hypothesis regarding placental metabolic changes leading to SPTB has received deeper investigation and recognition 157. A metabolomics analysis found significant changes in pathways and upstream regulatory components associated with inflammatory responses, mitochondrial function, redox status, signaling, energy metabolism, and homeostasis in SPTB placentas 158. This suggests that the placenta's insufficient ability to maintain bioenergetic and metabolic stability throughout pregnancy may eventually contribute to SPTB 158.

Bioinformatics analyses have emphasized the importance of FAs in PTB, highlighting that pathways such as fatty acid metabolism and lipid peroxidation are enriched in cases of PTB 159. Notably, FAO is significantly impaired in SPTB placentas, with elevated levels of various 2-hydroxy long-chain fatty acids, acylcarnitines, and PGs detected 158. Impaired FAO can lead to increased levels of free LCFAs and acylcarnitines, where excessive FAs serve as substrates for lipid peroxidation or directly participate in regulating inflammatory responses, contributing to SPTB. The accumulation of acylcarnitines can induce mitochondrial dysfunction and further inhibit FAO, potentially working in conjunction with abnormal FA metabolism to incite persistent inflammatory responses and oxidative stress, ultimately leading to placental dysfunction and/or membrane rupture, resulting in SPTB 160.

Inflammation has become a forefront topic in the pathophysiology investigation of PTB 161. Labor is a highly coordinated process characterized by synchronized cervical contractions, cervical ripening and dilation, and membrane remodeling and rupture, with inflammatory responses playing a crucial role 162. Intrauterine inflammation may arise from microbial infections or sterile inflammation within the amniotic cavity, which may occur without pathological infection 163. Sterile inflammation is significantly more severe in women experiencing preterm labor with intact membranes 163. Therefore, even without an infection, atypical inflammation might prematurely activate the key components, resulting in labor and PTB 164. In simplified terms, local pro-inflammatory factors induce PG production in uterine tissues, where PGE2 and PGF2α can promote cervical ripening and stimulate uterine contractility, thereby triggering PTB 165, 166.

Placental oxidative stress (OS) is another central pathological mechanism of preterm birth. In addition to the most common inflammatory causes, fatty acids significantly contribute to inducing placental OS. Specifically, ROS-induced DNA damage accompanied by telomere shortening promotes accelerated senescence of fetal membrane cells and induces inflammatory processes, directly leading to PTB 24. A non-targeted metabolomics study of plasma from mothers with preterm birth revealed an excess of unsaturated fatty acids, which predisposes to lipid peroxidation. Lipid peroxidation exacerbates maternal oxidative stress levels, leading to PTB by regulating cervical maturation, uterine contractions, and membrane rupture 159.

Mounting evidence suggests that DHA or a combination of DHA and EPA can prevent spontaneous or early PTB 167. The intake of DHA and EPA can inhibit PGs production, primarily PGE2 and PGF2α, as mentioned above. N-3 PUFAs and their derived mediators, such as SPMs, have important functions in suppressing inflammation, with mechanisms detailed in earlier sections regarding embryo implantation. Additionally, n-3 PUFAs may delay gestation through several mechanisms: a) n-3 PUFAs possess angiogenic properties that can enhance the development of capillary sprouts and hemodynamics, thus improving conditions related to placental developmental defects causing PTB 168; b) DHA can significantly inhibit the conversion of phosphatidylinositol and the mobilization of Ca2+ in response to oxytocin stimulation, as well as the membrane concentration of oxytocin receptors 169; c) the onset of labor is linked to increased expression of contraction-related proteins, activation of certain ion channels, and upregulation of connexin 43, all of which promote the electrical synchronization and coordination of uterine myometrial contractions. When contraction-related protein receptors are activated, the uterus contracts through oxytocin and PGs such as PGE2 and PGF2α. The physical properties of n-3 PUFAs lipid membranes may influence the activity of contraction-related protein receptors 170, 171.

### Gestational Diabetes Mellitus

Gestational diabetes mellitus (GDM) refers to women who develop abnormal glucose metabolism during pregnancy despite having normal glucose metabolism before. GDM is usually diagnosed in the second and third trimesters and is characterized by hyperglycemia and hyperinsulinemia accompanied by insulin resistance 172. The specific pathophysiological mechanisms underlying GDM remain unclear. The prevailing view is that its pathogenesis is similar to T2DM, characterized by increased insulin resistance (IR), decreased β-cell mass, and impaired function, leading to relative insulin deficiency 173. In fact, GDM is often regarded as the most prevalent metabolic disorder during pregnancy, frequently accompanied by elevated blood glucose levels, dyslipidemia, and heightened inflammation, particularly in the later stages of pregnancy 174.

It is widely recognized that the development of IR and GDM is substantially influenced by cytokine-induced chronic inflammation and adipokines 175. Compared to normal pregnancies, women with GDM exhibit elevated circulating levels of pro-inflammatory cytokines and leptin, while levels of adiponectin (an insulin-sensitizing adipocyte-specific factor) and anti-inflammatory cytokines are reduced, regardless of body mass index 176. The imbalance between pro-inflammatory and anti-inflammatory cytokines promotes the development and progression of IR and GDM. FAs may exacerbate or reverse IR by modulating inflammatory responses 173. In the blood of GDM women, levels of n-6 and n-3 PUFAs are elevated and reduced, respectively, suggesting that a high ratio of n-6/n-3 PUFAs may aggravate inflammatory responses 177. In contrast, SFAs (such as palmitic acid) and n-6 PUFAs are viewed as pro-inflammatory molecules 178. When acting as pro-inflammatory agents, FFAs can decrease the activity of phosphoinositide 3-kinase (PI3K) associated with insulin receptor substrate-1 (IRS-1) and inhibit the tyrosine phosphorylation of IRS-1, inducing IR in multiple organs, including skeletal muscle, pancreas, gastrointestinal tract, liver, and adipose tissue 178, 179. In GDM women, the intake of n-3 PUFAs has been shown to help alleviate IR and inflammation, thereby reducing the risk of adverse pregnancy outcomes 51, 178. Additionally, both maternal and placental OS are present in GDM. Placental OS increases inflammatory cytokines, particularly in cases where maternal BMI is elevated 24.

Although the prevailing consensus does not consider abnormal FAO to be a significant factor in the development of GDM, we have still found clues in some studies. Impaired FAO and mitochondrial overload can lead to acylcarnitine metabolic imbalances, disrupting insulin signaling through the regulation of mTOR phosphorylation and contributing to the onset and progression of IR and GDM 180. However, abnormalities in FAO are more likely to be secondary to restricted glucose utilization or inflammatory environments rather than being primary initiating factors. Research indicates that elevated levels of pro-inflammatory cytokines, particularly IL-6, in mothers with GDM lead to a significant decrease in the rate of FAO and an accumulation of excess fatty acids in the form of triglycerides (TG), resulting in placental lipotoxic damage 174. Furthermore, while elevated levels of FAs can be detected in GDM women, the consensus on whether GDM is induced by lipotoxicity remains inconclusive and requires further investigation 176, 181.

## Dietary Intervention

As previously mentioned, LC-PUFAs and their metabolites are essential for multiple physiological processes during pregnancy, such as controlling oxidative stress, angiogenesis, and inflammation. Furthermore, numerous pregnancy complications are often linked to decreased maternal LC-PUFAs levels or impaired placental FAs transport functions, potentially leading to a deficiency of essential LC-PUFAs for fetal growth and development. Consequently, the potential benefits of dietary LC-PUFAs for preventing or improving pregnancy complications have garnered increasing attention and research.

High-quality evidence indicates that consuming sufficient amounts of n-3 PUFAs during early pregnancy is associated with a reduced risk of PTB and early PTB in women with singleton pregnancies 182. Given that inflammation is a significant factor that causes preterm delivery, n-3 PUFAs may have a vital role because of their anti-inflammatory capabilities. A clinical consensus published in 2023 in the AJOG & MFM journal recommends that women of childbearing age should consume at least 250 mg/d of DHA + EPA from dietary sources, with an additional intake of ≥100-200 mg/d of DHA during pregnancy 167. Pregnant women with low DHA intake and/or low blood DHA levels are advised to consume approximately 600-1000 mg/d of DHA + EPA or to use DHA alone. It is preferable to implement this standard starting in mid-pregnancy, no later than around 20 weeks of gestation, and to continue until delivery or approximately 37 weeks 167. Women with normal DHA levels who satisfy their daily DHA requirements may not benefit from further DHA supplementation. It could even raise the risk of preterm birth 182.

Previous studies have found that the levels of DHA in the umbilical cord blood and maternal plasma of fetuses with FGR decline during pregnancy. At the same time, the expression of placental lipoprotein lipase, FABP1/3, and FATP1/2/4 increases 151. As previously discussed, this phenomenon may represent a compensatory response, although such compensation may be insufficient to reverse the DHA deficiency. Moreover, it remains unclear whether the decline in maternal DHA levels is a cause or result of FGR, with the possibility that both could be present. A meta-analysis indicated that supplementation with n-3 PUFAs during pregnancy does not reduce the risk of SGA or FGR 9. However, high-quality evidence suggests that supplementation with n-3 PUFAs may benefit certain anthropometric measurements in infants, such as low birth weight, head circumference, and length at birth (1-7 years) and birth weight, length, fat mass, and waist circumference 183. This effect could be attributed to the positive effects of n-3 PUFA supplementation on lengthening gestation (1-2 days) and affecting endogenous prostaglandin metabolism 182, 183.

Multiple studies have shown that plasma and umbilical cord blood levels of n-3 PUFAs are reduced in PE patients 166, 184, while strong evidence suggests that dietary supplementation with n-3 PUFAs can protect low-risk pregnant women from developing PE 185. Exogenous n-3 PUFAs may improve the oxidative stress and inflammatory status experienced by PE patients. Additionally, n-3 PUFAs may enhance the expression of FATP1 and FATP4 in the placental tissue of PE patients through the PPAR-γ pathway. This promotes DHA transport and improves endogenous n-3 PUFA levels in PE patients 186.

N-3 PUFAs can potentially prevent and treat GDM by decreasing the release of inflammatory cytokines and correcting glucose intolerance. Nevertheless, a clinical study conducted using a double-blind, randomized controlled trial demonstrated that adding DHA-rich cod liver oil does not have a preventive effect on the development of GDM 187. In GDM patients, combined supplementation with vitamin D and n-3 PUFAs for six weeks also positively impacts fasting blood glucose, serum triglycerides, very low-density lipoprotein cholesterol, and insulin-related indices 188.

In summary, the appropriate supplementation of n-3 PUFAs during pregnancy has become an essential component of nutritional intervention in recent years. However, the literature regarding the effects of n-3 PUFAs supplementation on neonatal outcomes and pregnancy complications remains inconclusive and contradictory, lacking further large-scale clinical studies and high-quality evidence. Further large-scale randomized controlled trials are necessary to determine the effects of variations in different n-3 PUFAs formulations, dosages, and intervention durations on neonatal outcomes and the incidence of pregnancy complications 189. Furthermore, it is necessary to consider the initial levels of DHA and EPA in both the mother and fetus when considering the potential preventive or therapeutic benefits of n-3 PUFAs supplementation 167. Crucially, for mothers with certain pregnancy complications, even if their DHA levels are normal or they are meeting the recommended daily intake of DHA, their offspring may still be deficient in DHA due to placental transport barriers. Defects in the transportation of DHA may cause this deficiency in fetuses through the placenta, which could explain why supplementing with n-3 PUFAs does not improve pregnancy outcomes in these cases 8. In such cases, although high-dose DHA supplements may not be effective in treating complications, they could help prevent fetal exposure to low levels of DHA by further increasing maternal DHA levels and improving placental fatty acid transport 8. It is also advisable to measure pregnant women's plasma n-3 PUFAs levels before starting supplementation, paying attention to minimizing potential harm to those with high baseline levels 167.

**Table 2** lists the recommended intake levels of n-3 PUFAs during pregnancy according to various professional organizations. While many organizations recommend taking n-3 PUFAs during pregnancy, most consider it an important nutrient rather than associating n-3 PUFAs supplementation with improved pregnancy complications or outcomes 167. However, a clinical practice guideline published in 2023 in the AJOG & MFM journal, adopted by academic groups across Europe, authoritatively indicated that supplementation with n-3 PUFAs can reduce the risk of PTB, emphasizing the need for earlier and greater supplementation of n-3 PUFAs for high-risk pregnant women with low DHA intake and/or low blood DHA levels 167. Future large-scale clinical studies are still needed to evaluate the minimum effective dosage range of n-3 PUFAs, the optimal combination, and the potential interactions of n-3 PUFAs with increasingly common interventions (such as aspirin and progesterone for reducing PTB) 167, 182. We eagerly anticipate the emergence of additional robust evidence and correlations concerning the advantages of n-3 PUFAs supplementation for improving pregnancy problems and outcomes.

## Conclusion and Outlook

In summary, FA metabolism is closely related to all stages of pregnancy, starting from embryo implantation. FAs and their metabolites maintain normal pregnancy through biological functions such as energy supply and regulation of oxidative stress and inflammatory states. It is noteworthy that although the mechanisms underlying the majority of pregnancy complications are complex and intricate, many of these pathways are initiated by placental dysfunction, with excessive oxidative stress and inflammatory states serving as core elements. Fetal growth restriction, preeclampsia, spontaneous preterm birth, and miscarriage may reflect a continuum of placental dysfunction 158. Encouragingly, FA metabolism can promote the normal growth and development of the placenta and fetus by providing oxidative energy, regulating various physiological processes, and expressing several important genes. Therefore, its significance is evident. A comprehensive understanding of the molecular mechanisms of fatty acid metabolism during pregnancy and the relationship between abnormal fatty acid metabolism and pregnancy complications is crucial for deepening our understanding of the importance of metabolism in female reproductive events. This understanding aids in viewing the three major metabolic processes organically and provides a theoretical basis for formulating corresponding clinical intervention measures.

By optimizing the intake of n-3 PUFAs, it may be possible to improve pregnancy outcomes and promote maternal and infant health, offering new directions for future reproductive medicine research. Subsequent studies should continue to explore the relationship between FA metabolism and pregnancy health, aiming to provide more comprehensive support and guidance for women's reproductive health.

## Figures and Tables

**Figure 3:**
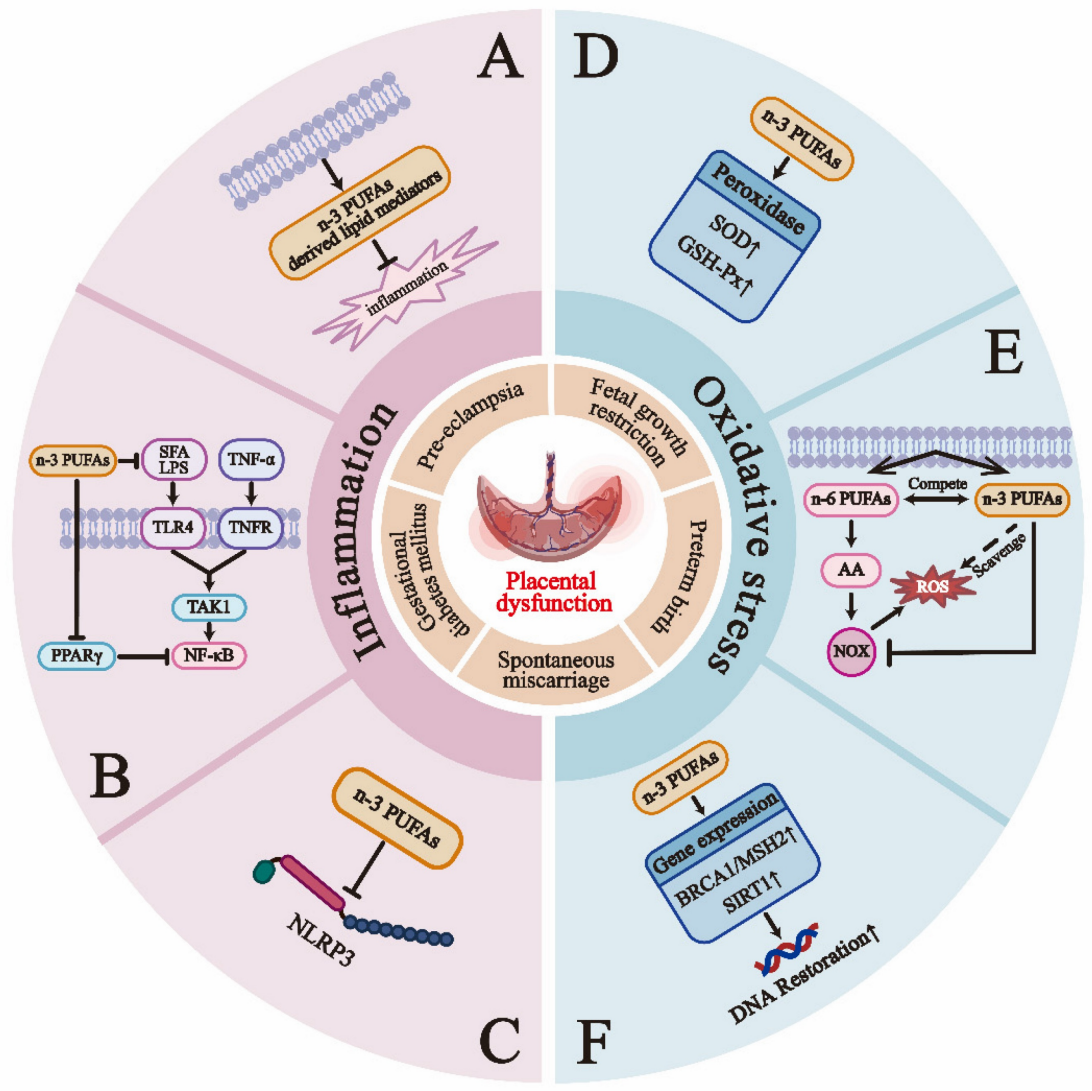
Gestational complications reflect the continuity of placental dysfunction, and n-3 PUFAs may improve pregnancy outcomes through their anti-inflammatory and antioxidant effects. The primary mechanisms of the anti-inflammatory action of n-3 PUFAs include: A) N-3 PUFAs produce SPMs via the LOX and COX pathways 53; B) N-3 PUFAs inhibit the phosphorylation of the inhibitory subunit IκB of NFκB, leading to reduced activation of the pro-inflammatory transcription factor NFκB. N-3 PUFAs also compete with lipopolysaccharides (LPS) and saturated fatty acids (SFA) to bind and activate toll-like receptors, such as TLR-4, inhibiting the activation of NF-κB 199; C) N-3 PUFAs suppress the activation of the NLRP3 inflammasome through GPR40 and GPR120-dependent pathways 52. Research on the anti-oxidant mechanisms of n-3 PUFAs is relatively limited, but can currently be summarized as follows 191, 192, 200: D) N-3 PUFAs can improve the status of peroxidases, such as increasing the activity of superoxide dismutase (SOD) and glutathione peroxidase (GSH-Px); E) N-3 PUFAs can lower levels of phagocyte and tissue-specific NADPH oxidases (NOX), which are significant contributors to ROS production. N-3 PUFAs may also inhibit NOX generation by competing to reduce the synthesis of AA, a primary activator of NOX; F) DHA can enhance the expression of antioxidant-related genes (such as SIRT1 and BRCA1/MSH2), which are crucial for DNA repair.

**Table 1:** Summary of the key functions of the two major categories of PUFAs during pregnancy.

Categories of PUFAs	Key Functions during Pregnancy	References
**N-3 PUFAs**	N-3 PUFAs and their derivatives exhibit potent anti-inflammatory properties	7, 50-52, 190
	Reduce the level of oxidative stress	191, 192
	Promote fetal growth and reduce the risk of low birth weight infants	9, 94
	Stimulate angiogenesis in the endometrium	77, 78
	Promote the development of the nervous system and retina, and enhance cognitive function	93, 193
	Improve insulin resistance	51
**N-6 PUFAs**	Regulate immune and inflammatory responses, primarily exerting pro-inflammatory effects, while lipoxins can exhibit anti-inflammatory properties	16, 194
	The derivatives 2-series PGs regulate the process of embryo implantation and uterine contractions	58, 195
	Promote fetal weight gain and the proliferation of adipose tissue	196, 197
	AA promote brain development by regulating cell division and signaling processes	198

**Table 2:** Recommended Intake Levels of n-3 PUFAs During Pregnancy

Organization (Year)	Dose (Per Day)	
	Pregnant women	Pregnant women with low DHA intakes or blood levels at the beginning of pregnancy
International Society for the Study of Fatty Acids and Lipids (2004)	≥200mg DHA	—
Perinatal Lipid Intake Working Group (2007)	≥200mg DHA	—
Food and Agriculture Organization of the United Nations (2010)	≥200mg DHA toward total 300mg EPA+DHA	—
European Food Safety Authority (2010)	250mg DHA+EPA and additional 100-200mg DHA	—
AFFSA, France (2010) and ANSES, France (2011)	250mg DHA/ 500mg DHA+EPA	—
Chinese Nutrition Society (2014)	250mg EPA+DHA, of which 200 mg should be DHA	—
American Journal of Obstetrics & Gynecology MFM, 2023	≥250mg DHA+EPA and additional ≥100-200 mg DHA	600-1000mg DHA+EPA, or DHA alone, preferably beginning in the second trimester of pregnancy and not later than approximately 20 weeks; Continue until childbirth or approximately 37 weeks
